# Sphingolipids in Hematopoiesis: Exploring Their Role in Lineage Commitment

**DOI:** 10.3390/cells10102507

**Published:** 2021-09-22

**Authors:** Yasharah Raza, Huda Salman, Chiara Luberto

**Affiliations:** 1 Department of Pharmacological Sciences, Stony Brook University, Stony Brook, NY 11794, USA; yasharah.raza@stonybrook.edu; 2 Renaissance School of Medicine, Stony Brook University, Stony Brook, NY 11794, USA; huda.salman@stonybrookmedicine.edu; 3 Department of Physiology and Biophysics, Stony Brook University, Stony Brook, NY 11794, USA

**Keywords:** sphingolipids, hematopoiesis, ceramide, sphingosine-1-phosphate, hematopoietic stem cells, erythrocytes, megakaryocytes, lineage commitment, myeloid differentiation, lymphoid differentiation

## Abstract

Sphingolipids, associated enzymes, and the sphingolipid pathway are implicated in complex, multifaceted roles impacting several cell functions, such as cellular homeostasis, apoptosis, cell differentiation, and more through intrinsic and autocrine/paracrine mechanisms. Given this broad range of functions, it comes as no surprise that a large body of evidence points to important functions of sphingolipids in hematopoiesis. As the understanding of the processes that regulate hematopoiesis and of the specific characteristics that define each type of hematopoietic cells is being continuously refined, the understanding of the roles of sphingolipid metabolism in hematopoietic lineage commitment is also evolving. Recent findings indicate that sphingolipid alterations can modulate lineage commitment from stem cells all the way to megakaryocytic, erythroid, myeloid, and lymphoid cells. For instance, recent evidence points to the ability of de novo sphingolipids to regulate the stemness of hematopoietic stem cells while a substantial body of literature implicates various sphingolipids in specialized terminal differentiation, such as thrombopoiesis. This review provides a comprehensive discussion focused on the mechanisms that link sphingolipids to the commitment of hematopoietic cells to the different lineages, also highlighting yet to be resolved questions.

## 1. Introduction

### 1.1. Hematopoietic Lineage Commitment

**Figure 1:**
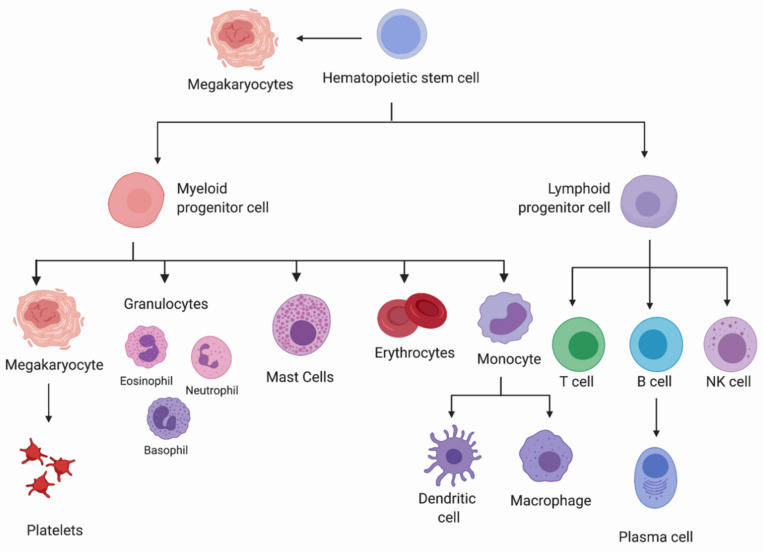
The Hematopoietic Differentiation Scheme. This schematic reflects the current understanding of the hematopoietic lineage commitment as a hierarchy, as well as a two-tier process of multipotent hematopoietic stem cells differentiating along a continuum into the megakaryocytic lineage. Created with BioRender.com (accessed on 28 July 2021).

Hematopoietic stem cells (HSCs) play a central role in maintaining life-long hematopoiesis and are characterized by three functional hallmarks: self-renewal, multipotency, and capacity to remain quiescent for long periods of time. HSCs reside at the apex of a hierarchical tree-like model of hematopoiesis (Figure 1) [1]. When an HSC undergoes lineage commitment, a cellular decision has been made to generate downstream progeny with restricted maturation potential. Lineage commitment is a fate decision whereby hematopoietic stem and progenitor cells (HSPC) have restricted lineage potential even when stimulated by growth factors that promote cells of other lineages to survive and proliferate [2].

HSCs have the ability to differentiate into any of the hematopoietic cell types, known as multipotency [3]. Differentiation has been traditionally understood as a stepwise process in which HSCs increasingly lose their self-renewal potential, and progenitor cells are successively generated, ultimately resulting in the derivation of mature blood cell lineages, including megakaryocytes, erythrocytes, mast cells, myeloblasts, and lymphocytes. However, as more sophisticated methodologies have been developed and applied (i.e., transcriptomics and epigenetics), this step-by-step view has been refined. For instance, recent studies have revealed profound transcriptional and epigenetic heterogeneity in HSCs at the single-cell level and pointed to the continuous, in addition to step-wise, nature of early hematopoietic commitment [4,5]. Furthermore, mechanisms that bypass the prerequisite passage through common multipotent progenitors have been identified with subsets of HSCs that may be inherently biased uniquely towards the megakaryocytic lineage [6,7,8,9,10,11]. These advances added a new “two-tier” approach to the classical hierarchical differentiation [8].

As differentiation is triggered and specific transcriptional programs are activated, important growth factors and cytokines influence the lineage commitment of these cells, including colony-stimulating factors, erythropoietin, interleukins, and/or thrombopoietin [12]. These factors are provided by stromal cells as well as by the hematopoietic cells, and combinations of these different factors have been shown to influence subsequent lineage commitment. As the focus of this review is the role of sphingolipids in hematopoiesis, herein we have presented only a very simplified view of how hematopoiesis is currently understood; this is intended as a guide for the analysis summarized in the later sections. For an in-depth discussion of the processes that govern hematopoiesis, the reader is referred to the following excellent reviews [4,11,13,14,15,16].

### 1.2. An Overview of Sphingolipid Metabolism

Sphingolipids and their function in the hematopoietic system have been irrevocably interlinked since the pioneer finding that sphingosine regulates apoptosis in HL-60 promyelocytic leukemia cells [17]. In addition to their role in apoptosis, sphingolipids play a critical role in other regulatory pathways, such as cellular differentiation, cell fate, growth, senescence, and more, as well as providing structural integrity to cells [18,19,20,21,22]. Certain players in the sphingolipid network are considered bioactive sphingolipids, in the sense that they trigger downstream signaling events. This includes (but is not limited to) ceramides, sphingosine, ceramide-1-phosphate (C1P), sphingosine-1-phosphate (S1P), and some classes of glycosphingolipids. As the levels of these bioactive sphingolipids are modulated by specific enzymes, the mechanism of regulation of these enzymes is also an important aspect to consider when studying sphingolipid-mediated signaling.

**Figure 2:**
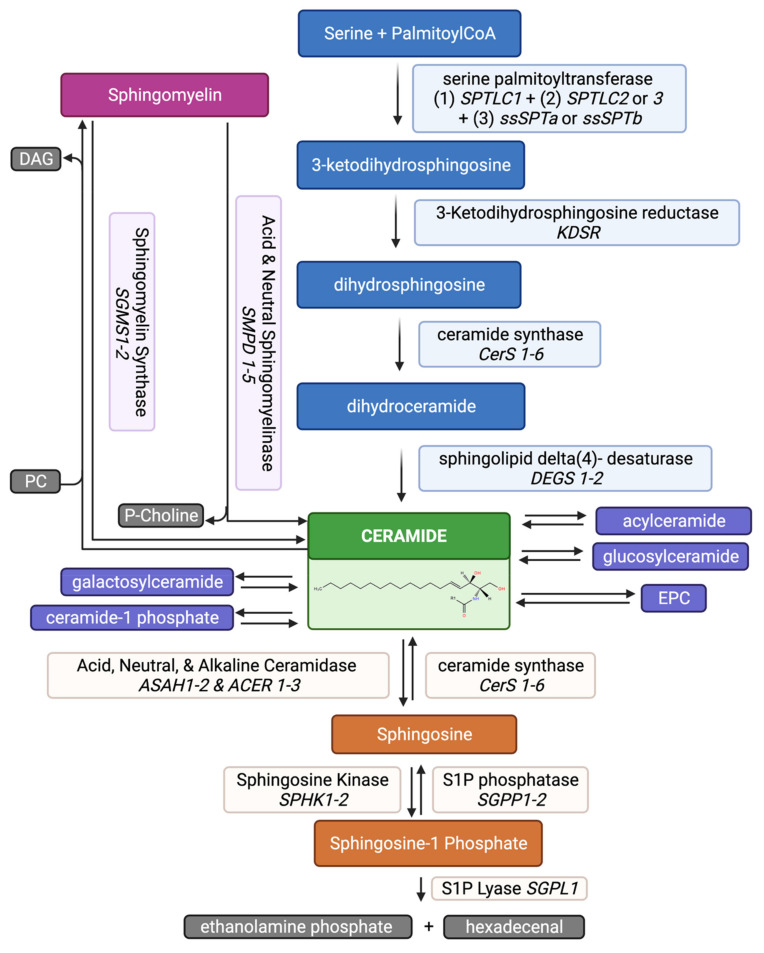
Sphingolipid Metabolism. In this simplified schematic overview of the sphingolipid pathway, gene names are italicized. The ceramide structure is from www.lipidmaps.org (accessed on 28 July 2021). Enzymes shown are either directly involved in hematopoiesis and/or lineage differentiation or they regulate a sphingolipid that is being discussed in the context of hemopoiesis. The figure was created using BioRender.com (accessed on 28 July 2021). PC: phosphatidylcholine; P-Chol: phosphoryl choline; R1: fatty acid; SPT: Serine Palmitoyl Transferase; SPTLC: SPT long base chain subunit; ssSPT: small subunit of SPT. SPT consists of three enzymatic subunits: SPTLC1, SPTLC2 or 3, and ssSPTa or b and one of three regulatory subunits ORMDL1-3.

Sphingolipids are found in nearly all plants and animals and are characterized by the presence of a sphingoid base backbone. De novo generation of sphingolipids starts with the condensation primarily (but not exclusively) of palmitoyl Co-A and L-serine by the enzyme serine palmitoyl transferase (SPT) [23,24,25,26] (Figure 2). The 3D structure of SPT revealed that the enzyme is a dimeric complex with each monomer composed of four subunits. In mammals, SPT long base chain subunit 1 (SPTLC1) together with SPTLC2 or SPTLC3 constitutes the catalytic core of the SPT enzymatic complex while a third subunit, a small subunit of SPT (ssSPT) a or b, stimulates the SPT activity and contributes to acylCoA substrate preference; finally, one of three regulatory subunits named ORMDL1-3 is also part of the monomeric complex [27,28,29]. Following the SPT-regulated first rate-limiting step, the 3-keto-dihydrosphingosine reductase (KDSR) is responsible for reducing 3-keto-dihydrosphingosine to dihydrosphingosine, which continues on to be acylated by ceramide synthase (CerS) and becomes dihydroceramide [30,31]. Dihydroceramide is in turn metabolized into ceramide upon the action of delta 4-dihydroceramide desaturase (DEGS) [32]. Ceramide lies at the center of the sphingolipid metabolic network as its metabolism branches out into several different routes. Ceramide can be: (1) converted into sphingomyelin (SM) by sphingomyelin synthase (SMS) with the transfer of a phospho-choline moiety from phosphatidylcholine (PC) onto the primary hydroxyl group [33] or into ethanolamine phosphoceramide (EPC) by the transfer of phospho-ethanolamine from phosphatidylethanolamine (PE) [34,35]; (2) glycosylated at the same primary hydroxyl group by glucosylceramide synthase (GCS) or by galactosylceramide synthase (GalCerS) with the addition of glucose or galactose, respectively [36,37]; these hexosylceramides will go on to form more complex sphingolipids with the addition of additional sugars and sialic acids to synthesize gangliosides; (3) phosphorylated by ceramide kinase (CERK) to generate ceramide-1-phosphate (C1P) [38]; (4) acylated either via the concerted action of fatty acyl CoA synthase (ACSL5) and diacylglycerol acyltransferase 2 (DGAT2) [39] or by the activity of PNPLA1 in the skin [40]; (5) hydrolyzed by ceramidase (CDase) to cleave the fatty acid and generate sphingosine [41]. Sphingosine can then be phosphorylated into sphingosine-1-phosphate (S1P) by the sphingosine kinase (SPHK) [42] or it can revert back to ceramide by the action of CerS [43]. The catabolism of all sphingolipids ultimately occurs via their conversion into sphingosine and then S1P which in turn is broken down by the S1P lyase into hexadecenal and ethanolamine phosphate. This is the only known way to exit the sphingolipid metabolism.

Relevant to S1P functions in hemopoiesis is the ability of S1P to be transported extracellularly and to activate in an autocrine or paracrine fashion five different S1P receptors, S1PR_1–5_ (formerly known as Edg receptors). S1PRs are G-protein coupled receptors present in a variety of cell types with specific patterns [44,45,46]. They are generally highly expressed on leukocytes and while S1PR_1–3_ is expressed ubiquitously, S1PR_4_ is only expressed on lymphatic and hematopoietic tissues [47,48,49].

Alterations in sphingolipid metabolism have been implicated in many common human diseases, including hematological malignancies (for comprehensive reviews on this topic the reader should refer to [18,50,51,52,53]). Interestingly, bioactive sphingolipid metabolism has also begun to emerge as an important factor in normal hematopoiesis. It is crucial to develop an understanding of how sphingolipids function in normal hematopoiesis to also appreciate how their dysregulation is implicated in hematological malignancies. Paradoxically, the understanding of the roles and functions of sphingolipids in normal hematopoiesis is still in its initial phases, with recent observations suggesting yet unrecognized roles for this class of lipids. No review to date has comprehensively discussed the functions of sphingolipids and sphingolipid-metabolizing enzymes in normal hematopoiesis from HSCs all the way to mature lineages. The sections that follow are a comprehensive discussion of this topic organized by the different hematological lineages and with the discussion of still unresolved questions or contentious conclusions. The focus is mostly on the path to terminal differentiation.

## 2. Sphingolipids in Hematopoiesis

### 2.1. Sphingolipids and Hematopoietic Stem Cells

Very few studies have explored the effect of sphingolipids on the modulation and mobilization of HSCs, hence, our current understanding of the role of sphingolipids in the regulation of HSCs remains limited. Initially, a study by Maguer-Satta et al. suggested a negative role for ceramide in maintaining cellular stemness. Treatment with ceramide analogs (C2 or C6-short chain ceramides) produced similar effects to tumor necrosis factor (TNF) in inhibiting the ability of long-term culture-initiating cells isolated from human CD34^+^CD38^−^ cells to produce delayed colony-forming cells [54]. While this inhibitory effect was independent of cytotoxicity and was specific to these long-term culture-initiating cells (as opposed to short-term culture-initiating cells), the biological relevance of these observations remains in question as the functional relevance of these in vitro derived long term culture-initiating cells is unclear.

More recently, the link between sphingolipids and stemness was further explored [55]. First, it was found that specific patterns of the sphingolipidome characterize HSCs, progenitors, and terminally differentiated hematopoietic cells. In particular, different levels of ceramide/dihydroceramide ratios were found among the different hemopoietic cell types, with increased levels of specific dihydroceramide species in stem versus progenitor cells. These different lipid patterns raised the question of the role of ceramide homeostasis and ceramide-regulating enzymes in self-renewal and lineage commitment [55]. Thus, further investigations revealed that the metabolic step that converts dihydroceramide into ceramide plays a crucial role in commitment to lineage differentiation in response to stress-induced activation. In fact, when ceramide formation was inhibited by blocking DEGS1 during the transition of HSCs from quiescence to activation, stress adaptation pathways that contribute to maintaining stemness, such as ER stress and autophagy, were activated [55]. Given the accumulation of specific dihydroceramide species in quiescent HSCs as compared to progenitors and that inhibition of DEGS1 in activated HSCs caused the accumulation of dihydroceramide while preserving stemness, a critical function for dihydroceramide/ceramide homeostasis in stemness is likely.

In addition to intrinsic roles for ceramide metabolism, paracrine roles for sphingosine-1-phosphate (S1P) in the mobilization of HSCs via the engagement of S1P receptor 1 (S1PR_1_) on HSCs were identified. HSCs normally reside in the stem cell niche within the BM and only a minimal fraction of HSCs is found in circulation. A high concentration of S1P in circulation is critical to favor the egress of HSCs from the stem cell niche into the blood and maintenance of an S1P gradient between the blood and the BM is essential for HSC mobilization [56,57]. Furthermore, S1PR_1_ on HSCs was identified as the receptor which senses S1P in circulation [56,58]. The functional inhibition of S1P receptors including S1PR_1_ with the sphingolipid analog FTY720 [59,60] or by using hematopoietic conditional S1PR_1_ knock out mice, led to reduced HSC mobilization following the blockage of CXCR4, the HSC receptor that tethers HSCs to the BM [56,58,61,62,63]. In a complementary approach, stimulation of S1PR_1_ with SEW2871 was shown to promote mobilization induced by the blockage of CXCR4 [58]. Based on these observations, activation of S1PR_1_ could represent a potential clinical strategy to improve HSC mobilization when using peripheral blood stem cells for transplantation. On the other hand, the S1P-S1PR_1_ axis was not involved in the mobilization of HSCs by G-CSF treatment. Similar to the egress of HSCs from the BM into the bloodstream, the egress of HSCs from peripheral organs into the lymphatic system seems also to be positively regulated by S1PR_1_ [64]. The treatment of mice with FTY720 consistently increased the number of HSCs in the tissues.

The stimulatory role of S1PR_1_ for the egress of HSCs from the BM came into question in another study showing that the treatment of mice with SEW2871 did not affect the mobilization of Kit^+^/Sca-1^+^/Lin^−^ hematopoietic progenitor cells after the blockage of CXCR4 [65]. Opposite to what was shown in the previous studies, pretreatment with the S1PR_1_ antagonist W146 favored mobilization of Kit^+^/Sca-1^+^/Lin^−^ progenitor cells in circulation. The reason for this discrepancy is not immediately clear, and this remains an area that requires further investigation.

### 2.2. Sphingolipids in Erythroid Differentiation and Erythrocytes

Erythrocytes, more commonly known as RBCs, contain hemoglobin which imparts a red color to the blood and are responsible for transporting oxygen throughout the body. After HSCs progressively differentiate into the common myeloid progenitors (CMP), CMPs give rise to the megakaryocyte-erythroid progenitor cells (MEP). As their name implies, MEPs can differentiate into megakaryocytes or erythrocytes. MEPs commit to erythropoiesis by differentiating into the burst forming unit-erythroid (BFU-E) progenitors and then into the colony-forming units-erythroid (CFU-E) progenitors [66]. In vitro, the formation of CFU-E colonies is dependent on erythropoietin [67], while in vivo, erythropoietin controls the number of CFU-E cells in the BM [68]. From CFU-E, the cells advance into the terminal stages of differentiation by a step-wise maturation process into proerythroblasts (ProE), basophilic erythroblasts (BasoE), polychromatophilic erythroblasts (PolyE), and orthochromatic erythroblasts (OrthoE) [69]. It is during this progression that the cells start accumulating hemoglobin. Upon enucleation, the cells mature into reticulocytes, and subsequently, upon loss of the other organelles and remodeling of the plasma membrane, they transform into erythrocytes [70].

A connection of sphingolipids in erythropoiesis was seen as early as 1974 when Clayton et al. proposed a link between bioactive sphingolipids and erythroid lineage commitment. Clayton et al. identified the ability of select ceramides with different fatty acyl chains (C24:0, C24:1- and to a smaller extent C22:0) and SM to induce erythropoiesis in rabbit BM cells [71]. The involvement of SM was further corroborated in 1986 by Scaro et al., when an extract of phospholipids from human plasma, as well as purified SM, were injected into mice. This resulted in an increased number of reticulocytes in circulation, suggestive of an increase in erythropoietic turnover [72]. Furthermore, Dallalio et al. showed that ceramide inhibited CFU-E colony formation while S1P reversed this inhibition [73]. Similarly, S1P counteracted the inhibitory effect of tumor necrosis factor-α (TNFα) and gamma interferon on erythropoiesis [73,74]. Thus, these studies indicate a function for S1P in erythroid differentiation.

Mechanistic insight into the function of S1P in erythropoiesis is provided by Yang et al. S1P signaling contributes to the modulation of mitophagy [75], an essential process by which reticulocytes eliminate mitochondria and control later stages of erythroid differentiation [76,77]. While the first stages of erythropoiesis are mostly regulated by factors controlling macro-autophagy (such as Atg5 and 7), later stages are accompanied by higher expression of mitophagic factors (such as Pink1 and Nix/Bnip31). This work uncovered that sphingolipid signaling impacts genes that regulate the latter process. Inhibiting SPHK1, one of the two isoenzymes producing S1P, led to decreased *PINK1* expression which was associated with impaired mitochondria clearance from the cells, impaired erythrocyte maturation, and cell death. By supplementing these cells with exogenous S1P, the authors were able to enhance normal erythroid maturation, revealing a role for S1P specifically in mitophagy during erythropoiesis. However, while the expression of SPHK1 increases during erythropoiesis [75], the absence of both SPHK1 and 2 in erythrocytes resulted in embryonic lethality due to defects in the vasculature and not because of major effects on the erythrocytes themselves [78]. In line with a marginal role for SPHKs in erythropoiesis, transplantation of HSCs isolated from SPHK1 and 2 double knock out mice in adult wild-type mice, had no effect on RBC development [78]. Thus, the role of S1P production in adult erythropoiesis is inconclusive with contradictory effects in these two studies [75].

**Figure 3:**
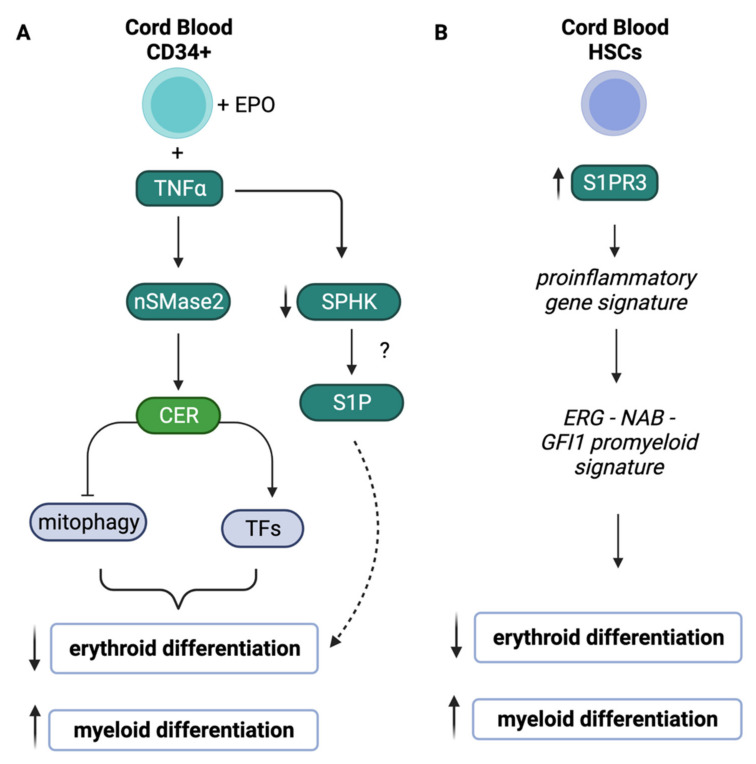
Roles of Sphingolipids and proinflammatory signals in erythroid and myeloid lineage commitment in CD34^+^ cells (**A**) and hematopoietic stem cells (HSCs) (**B**). Created with BioRender.com (accessed on 28 July 2021). EPO: erythropoietin, TF: transcription factors, TNFα: tumor necrosis factor alpha, nSMase2: neutral sphingomyelinase 2, SPHK: sphingosine kinase, S1P: sphingosine-1-phosphate, CER: ceramide, S1PR3: sphingosine-1-phosphate receptor 3, HSCs: hematopoietic stem cells.

The involvement of bioactive sphingolipids in erythropoiesis and autophagy has also been revisited in the context of pro-inflammatory signals by Orsini et al. [74] (Figure 3A). Ceramide was shown to inhibit erythropoiesis in human cord blood CD34^+^ hematopoietic stem/progenitor cells. In fact, treatment with C2 and C6 synthetic ceramides as well as bacterial sphingomyelinase (bSMase) inhibited erythroid differentiation induced by erythropoietin in vitro. Moreover, building on the functional link between ceramide and erythropoiesis, the authors also connected changes in ceramide caused by TNFα to the inhibition of erythropoiesis. TNFα is known to exert a negative effect on erythroid differentiation [79,80] and the investigators propose a role for ceramide produced by neutral sphingomyelinase 2 (nSMase2) in mediating this negative effect. Specific down-regulation/inhibition of nSMase2, in fact, restored erythropoietin-mediated differentiation otherwise hindered by TNFα. Further to this, it was shown that TNF or ceramide treatment induced the activity of specific transcription factors to promote a profile compatible with inhibition of erythropoiesis (increased levels of PU.1 and GATA2 and reduced activity of GATA1) positioning this mechanism of regulation downstream of ceramide. Mechanistically, ceramide treatment activated mTOR and inhibited ULK1, causing Atg13 dephosphorylation and leading to the inhibition of late stages of autophagy, a process required for erythropoiesis [81]. Notably, TNFα and ceramide treatment of CD34^+^ cells, by affecting the profile of the mentioned key transcription factors, not only inhibited erythropoietin-induced erythroid differentiation but also promoted granulomonocytic differentiation, essentially converting the expected process of erythropoiesis to myelopoiesis.

**Figure 4:**
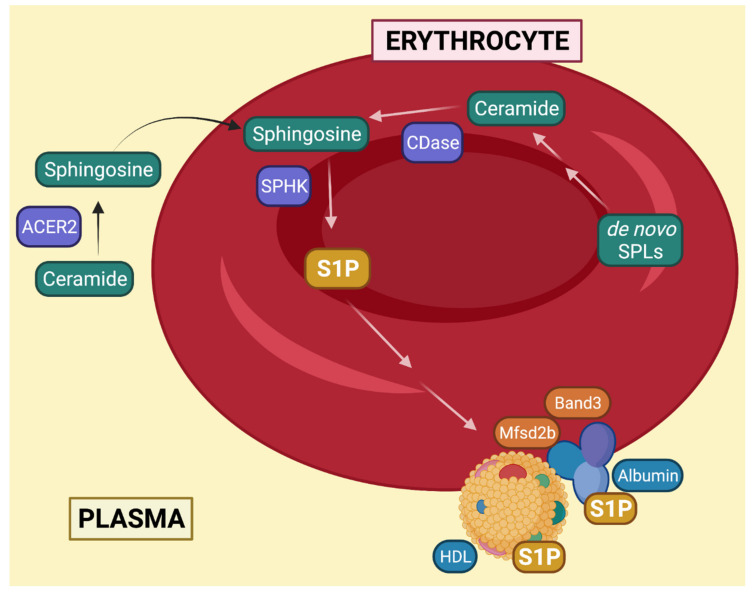
S1P production in erythrocytes. Synthesis of S1P in erythrocytes occurs from utilization of either plasma or intracellular sphingosine. Efflux of S1P is mediated by Mfsd2b, and Band3 and facilitated by HDL and albumin in the blood. Created with BioRender.com. SPHK: sphingosine kinase, CDase: ceramidase, SPL: sphingolipid, ACER2: alkaline ceramidase 2, S1P: sphingosine-1-phosphate, HDL: high-density lipoproteins.

The high level of S1P in the blood is critical to establish an S1P gradient that allows the S1PR_1_-mediated egress of HSCs from the BM into the blood and of lymphocytes from secondary lymphoid organs into circulation [82,83,84,85]. Together with vascular endothelial cells, RBCs are a major contributor of blood S1P and they store large amounts of this sphingolipid [78,82]. A major breakthrough in understanding the metabolic regulation of S1P in RBCs came from a study by Ito et al. who reported that these cells have negligible activity of the enzymes that degrade S1P, specifically S1P lyase and phosphatase [86]. This, together with substantial SPHK activity [87], favors the intracellular accumulation of S1P. Further studies have also clarified the source of sphingosine utilized to synthesize S1P in RBCs and found that sphingosine can be either directly taken up from the plasma or produced via de novo sphingolipid biosynthesis (Figure 4). In the case of plasma sphingosine, it was shown to derive from the hydrolysis of ceramide by ACER2, one of three alkaline ceramidases [88]. In fact, mice where ACER2 was knocked out in hematopoietic cells showed reduced blood levels of both sphingosine and S1P. The activity of a ceramidase must also be involved to generate intracellular sphingosine produced via de novo sphingolipid biosynthesis. Since sphingosine can only be generated by the hydrolysis of ceramide, it is hypothesized that ceramide synthesized following the desaturation of dihydroceramide is acted upon by a yet to be identified ceramidase [87]. Once formed in RBCs, S1P is released extracellularly via the activity of the Mfsd2b transporter (similarly to platelets) [87,89,90] and possibly through the action of the abundant erythroid transporter, Band3 [91]. Importantly, S1P efflux from RBCs is also facilitated by the presence of high-density lipoproteins and serum albumin [92,93,94]. In addition to releasing S1P into the plasma, RBCs can also directly deliver it into the tissues under specific circumstances. In fact, in case of tight contact between RBCs and endothelial cells (such as in capillaries and tissue interstitium), S1P can be transported transcellularly into the tissues [93].

In addition to providing S1P in RBCs for its release in circulation, SPHK1 activity in RBCs is required to channel glucose into the glycolytic pathway and enhance the production of 2,3-bisphosphoglycerate [95,96]. The 2,3-bisphosphoglycerate binds with a higher affinity to deoxygenated hemoglobin than to hemoglobin carrying oxygen, it stabilizes the low oxygen state of this carrier and ultimately favors the release of the oxygen in the tissues. This implies that SPHK1, by modulating 2,3-bisphosphoglycerate in RBCs, favors tissue oxygenation and could play a beneficial function in conditions that necessitate it, such as higher altitude [97].

In addition to S1P, changes in SM levels during erythroid differentiation are also linked to RBC functions. Human erythrocytes are enriched in SM [55] compared to other terminally differentiated hematopoietic cells, and recent lipidomics analysis of murine erythroblasts at different stages of differentiation has revealed a correlation between the progressive increase of sphingosine, SM, and to a certain extent, of ceramide with erythroid markers of differentiation [98]. The presence of SM in lipid microdomains in the outer leaflet of the plasma membrane of RBCs has been linked to the ability of these cells to dynamically change their shape [99]. The RBC plasma membrane is characterized by the presence of lipid microdomains of specific composition. Microdomains enriched in SM and cholesterol, as well as those enriched in ganglioside GM1 and cholesterol, are found at low curvature sites on the RBC plasma membrane. Recent studies have linked the modulation of these microdomains with changes in the intracellular calcium level and reshaping of RBCs [99,100].

### 2.3. Sphingolipids in Thrombopoiesis and Megakaryocytes

Thrombopoiesis is the process that leads to the production of platelets, first characterized by the formation of megakaryocytes (megakaryopoiesis) followed by further rearrangement of these cells into proplatelets, the platelets producing entities. Megakaryopoiesis occurs in the BM and as discussed earlier, is either initiated by the differentiation of HSCs into a specialized megakaryocytic unipotent subset of cells [6,7,8,9,10,11], or by HSC progression through the same differentiation steps as erythropoiesis until the cells reach the MEP stage, at which point, the two processes diverge, and MEPs go on to produce megakaryocytes. The final stage of thrombopoiesis is characterized by the formation of pro-platelets from megakaryocytes [101,102]. The formation of proplatelets is accompanied by endomitosis, mass increase, and major rearrangement of the cytoskeleton with protrusions extending into sinusoids [101,102]. Proplatelets are shed into circulation by these protrusions and in turn mature into platelets, possibly in part due to exposure to high levels of S1P (see below) [103].

Thrombopoietin is one of the critical cytokines that supports megakaryopoiesis and maintenance of the number of megakaryocytes and platelets. However, since cells retain megakaryopoiesis ability in the absence of thrombopoietin [104], it is thought that other factors also contribute to late differentiation and morphological changes during thrombopoiesis. Golfier et al. began to address this gap of knowledge and specifically investigated the role of sphingolipids in the later stage of megakaryocytic lineage commitment. They found that one of five S1P receptors [49,105,106,107,108,109,110], S1PR_4_ is upregulated during megakaryopoiesis [111]. While S1PR_4_ levels in CD34^+^ cells were undetectable, high S1PR_4_ expression was observed in CD41^+^ cells (megakaryocytes) and not in non-megakaryocytic cells. Different strains of mice that lacked the *S1PR_4_* gene mice were deficient in platelet repopulation and had significantly fewer pro-platelets. The overexpression of S1PR_4_ in human erythroleukemia (HEL) cells induced the formation of CD41^+^ like cells, with the same cytoplasmic rearrangement and pro-platelet formation as observed in normal megakaryopoiesis. Altogether, this evidence suggests S1PR_4_ supports the final steps of megakaryopoiesis [111].

Other studies support a role for extracellular S1P to regulate proplatelets shedding, however, the outcome and the exact mechanism of such regulation is controversial. Earlier evidence by Zhang et al. pointed to a stimulating function of blood S1P on megakaryocytes [103]. Specifically, blood S1P promoted the elongation of proplatelet extensions and shedding into the BM sinusoids via activation of S1PR_1_, and mice lacking the S1PR_1_ receptor developed thrombocytopenia [103]. However, more recently, this mechanism has come into question. In fact, mice with S1PR_1_ selective deletion in mature megakaryocytes did not show reduced platelet count nor S1PR_1_ seemed to be expressed or activated to detectable levels on the surface of megakaryocytes [112], disputing a role for the blood S1P-megakaryocytic S1PR_1_ axis in platelet shedding. Surprisingly, the specific deletion of S1PR_1_ in hematopoietic progenitor cells resulted in elevated platelet counts suggesting a rather negative role for this S1P receptor. A negative role of S1P and S1PR_2_ on megakaryopoiesis was reported as well [112,113]. In these studies, systemic loss of SPHK2 impaired the intravascular shedding of proplatelets. However, when loss of SPHK2 was selective to megakaryocytes, no effect on the number of platelets was observed [112], and the negative impact of the systemic loss of SPHK2 was traced back to increased extracellular S1P levels in these mice and activation of S1PR_2_ rather than an intrinsic function of SPHK2 in megakaryocytes.

Other studies have found that thrombocytopenia is associated with mutations of *KDSR* in four out of the eight cases in which *KDSR* mutations were reported [114,115]. In humans with *KDSR* mutations, the hyperproliferation of megakaryocytes and dysplasia were also reported [114,115]. Bariana et al. confirmed that mutant *KDSR* enhanced megakaryocytic proliferation and impaired pro-platelet formation by CD34^+^ cells in vitro [115]. Complementation with functional KDSR in deficient cells led to the rescue of the proplatelet phenotype and of the cytoplasmic organization. These results indicate that KDSR may play a critical role in the pro-platelet formation of megakaryocytes during differentiation.

Most recently, Xie et al. identified a novel role for S1PR_3_ in myelopoiesis and megakaryopoiesis [116]. While investigational efforts were more focused on the role of S1PR_3_ in leukemia and myelopoiesis, the study found that S1PR_3_ over-expression in human HSCs favored CD33^+^ myeloid and CD41^+^ megakaryocytic lineage commitment at the expense of erythroid and lymphoid lineage commitment [116]. This observation implicates S1PR_3_ in megakaryopoiesis. The specific effects of S1PR_3_ on myeloid differentiation will be further explored later in this review.

Platelets store considerable amounts of S1P [117] and the enzymatic routes leading to the formation of the platelet S1P have been thoroughly investigated. Mature megakaryocytes and platelets appear to have high levels of sphingosine kinase activities brought by SPHK1 and/or SPHK2 [113,118,119]. In platelets, the abundance of SPHKs together with the lack of one of the enzymes that degrade S1P, the S1P lyase [120], leads to high levels of intracellular S1P. Interestingly, de novo sphingolipid synthesis in platelets appears to be weak, and it is proposed that platelet S1P is formed from the phosphorylation of sphingosine taken up from the extracellular space or formed in the outer leaflet of the plasma membrane from hydrolysis of SM [121].

Despite the significant amount of intracellular S1P, in normal conditions, platelets do not contribute to plasma S1P, a function elicited by erythrocytes and endothelial cells [84,122]. On the other hand, platelets seem to control the S1P level in the serum [123] in response to specific stimuli such as thrombin [124] or fluid shear stress [125]. The release of S1P by activated platelets has been linked to the formation of atheromas. In fact, high levels of serum sphingoid base phosphates (S1P and dhS1P) are associated with thrombocythemia and are best correlated with platelet count rather than RBCs [126]. Recent studies have proposed different potential mechanisms for the release of S1P from platelets. First, the major facilitator superfamily transporter 2b (Mfsd2b) has been shown to export S1P from platelets and RBCs into the blood [89]. Consistently, platelets and RBCs from Mfsd2b knock out mice accumulated S1P intracellularly while its overexpression favored the active extracellular transport of S1P. In addition to Mfsd2b, the ATP-dependent transporter, Multidrug Resistance Protein 4 (MDRP4), has also been proposed to contribute in part to the release of S1P from platelets [127]. Platelets from mice lacking MDRP4 secreted less S1P following activation with the thromboxane receptor agonist, U-46619. MDRP4 facilitates the loading of S1P into storage granules which are secreted by platelets upon activation [128]. In line with an active role for MDRP4, the S1P level was found to be significantly higher in the serum from WT compared with MRP4^−/−^ mice [128]. Thus, altogether, these studies suggest that the release of S1P from activated platelets may occur via two mechanisms, one mediated by Mfsd2b at the plasma membrane and one mediated by MDRP4 from storage granules.

### 2.4. Sphingolipids in Myeloid Differentiation

Traditionally, myeloid cells have been thought to arise from the CMP. The CMP further differentiates into granulocyte/macrophage progenitors (GMP) which in turn commit to granulocytic (eosinophil, neutrophil, and basophil) or monocytic lineages [129]. Recently, this view has been revised to include two distinct pathways of myelopoiesis. Myeloid cells can be GATA1 positive (GATA1^+^) and that includes mast cells, basophils, and eosinophils, or GATA1 negative (GATA1^−^) with neutrophils, monocytes, and macrophages [130]. Based on this distinction, Drissen et al. sought to identify whether these two myeloid groups could be differentiated on the basis of lineage commitment. It was observed that CD34^+^CD38^+^ progenitors have the potential to develop neutrophils/monocytes or eosinophils/basophils/mast cells, and they cannot give rise to all of the myeloid cell types [131]. This indicates that GATA1^+^ and GATA1^−^ myeloid cells separate at an earlier stage than initially thought, and that there are two pathways of myelopoiesis.

Sphingolipid metabolism plays a role in promoting myelopoiesis. Expression of the S1P receptor, S1PR_3_ has been specifically associated with myeloid lineages; while *S1PR_3_* expression is very low in primitive CD34^+^ cells, it is detected on a small portion of granulocyte-monocyte progenitors and found to be specifically expressed in granulocytes and monocytes [116]. The role of S1PR_3_ in lineage commitment was directly evaluated by overexpressing S1PR_3_ in human HSCs; in this case, S1PR_3_ favored CD33^+^ myeloid and CD41^+^ megakaryocytic lineage commitment at the expense of erythroid and lymphoid lineages as observed in in vitro clonogenic assays and/or in vivo transplantation (Figure 3B) [116]. The overexpression of S1PR_3_ in HSCs also induced a pro-inflammatory gene response program (including NF-kB activated genes) and an ERG-NAB-GFI1 myeloid gene regulatory network. As this particular lineage profile mimics inflammation-induced changes [116], a cooperative effect between the expression of S1PR_3_ and TNFα was shown to potentiate myelopoiesis.

As we mentioned earlier, ceramide generated in response to TNFα via the action of neutral sphingomyelinase 2 was also shown to promote myelopoiesis while inhibiting erythroid differentiation [74]. This effect was associated with changes in specific transcription factors, such as increased levels of PU.1 and GATA-2 which are known to inhibit erythropoiesis and favor myeloid differentiation.

*Granulocytes:* Parthibane et al. identified a novel role for SPTLC1 in myeloid differentiation [132]. Specifically, the deletion of SPTLC1 in the BM yields significantly fewer granulocytes. SPTLC1 deletion did not affect erythroid or lymphoid output, nor the generation of MEPs. On the other hand, the generation of CMPs was significantly depleted, indicating an important role of SPTLC1 in granulopoiesis. Building upon these findings, this group continued their investigation of SPT in hematopoiesis, and they also revealed a role for ssSPTa in myelopoiesis similar to that of SPTLC1 [133]. Specifically, the loss of ssSPTa, but not of ssSPTb, from adult HSCs using Mx-1-Cre mice led to specific impairment of myelopoiesis with increased Lin^−^Sca1^+^c-Kit^+^ stem cells, as well as increased progenitors. [133]. Molecularly, the defect in myelopoiesis was accompanied by evidence of ER stress. Altogether, in vivo results in *SPTLC1* and *ssSPTa* conditional mouse models support a central role for SPT in myelopoiesis.

*Monocytes/Macrophages:* Several early studies utilized in vitro models of monocytic/macrophagic differentiation to probe sphingolipid changes during the process. For the most part, these models employed the treatment of specific cell lines such as HL-60, U937, and THP-1 with either vitamin D_3_ or the phorbol ester, TPA. Some consistent changes correlating with differentiations included the modulation of glycosphingolipids, albeit different classes of glycosphingolipids responded differently depending on the cell type and treatment. An increase of ganglioside GM3 and decrease of lactosylceramide was reported early on following the differentiation of HL-60 cells into macrophage-like cells with TPA, suggesting the stimulation of GM3 synthase [134,135,136]. The stimulation of GM3 synthase was indeed confirmed [137] and treatment of HL-60 and U937 cells with exogenous GM3 was shown to induce differentiation into monocyte/macrophage lineage [138,139]. The activation of GM3 synthase and increase of GM3 were also confirmed using HL-60 cells sensitive to TPA-induced differentiation versus those resistant to the treatment and in human blood monocytes [140,141]. In addition to GM3, GM1 has also been linked to monocytic differentiation and most interestingly, its high surface level defined a small subset of peripheral blood human monocytes which dramatically expanded in number upon in vitro differentiation into macrophages [142,143]. In some cell models, such as U937 and HL-60 treated with TPA, stimulation of the initial steps of the synthesis of glycosphingolipids was also observed with the increased activity of GCS and accumulation of glucosylceramide [144,145]. Similarly, an increase of SM synthesis also accompanied monocytic/macrophage differentiation and these complex sphingolipid changes were reflective of only the adherent portion of the differentiating cells [145,146]. Importantly, both the accumulation of glycosphingolipids and SM seems to contribute to adhesion of macrophage-like cells, as treatment with PDMP, an inhibitor of GCS, or exogenously added bacterial sphingomyelinase blocked adherence of the differentiated cells. In addition to SM, lysoSM was also found to promote the adhesion of TPA-treated U937 and THP-1 cells [147]. Changes in other sphingolipids have also been reported, such as C1P via stimulation of CERK activity in BM-derived monocytes [148] and ceramide via the hydrolysis of SM in HL-60 cells treated with vitamin D_3_ [149]. A recent report also detailed changes of some sphingolipids and sphingolipid-metabolizing enzymes in human peripheral blood monocytes during differentiation with human monocyte-colony stimulating factor [150]. While a decrease of total lactosylceramide confirmed previous reports, no significant changes in total levels of ceramide, SM, and hexosylceramide were observed. As results were reported only at day four of differentiation, additional dynamic changes at different times could have been missed. Furthermore, the levels of acid sphingomyelinase and acid ceramidase increased with differentiation possibly to push production of S1P and extend the life of macrophages.

In addition to sphingolipids, changes in the expression of specific S1PRs have also been documented. An early study probing mRNA expression by RT-PCR reported that human blood monocytes express mostly S1PR_2_ and S1PR_3_ [151] with little to no S1PR_1_. Further to this, when Hohenhaus et al. thoroughly characterized the expression of the G-protein coupled receptor (GPCR) by RT-PCR during monocyte to macrophage differentiation, they found that S1PR_1_ and S1PR_4_ seemed to have contrasting patterns, with low S1PR_1_ and high S1PR_4_ levels when differentiating from monocytes to macrophages [152]. Further to this, another study reported that the M1 specific polarization of murine BM macrophages led to the suppression of S1PR_4_ expression [153] suggesting that specific S1PR patterns on macrophage subtypes might be functionally linked to implementing specific processes (i.e., migration). For a comprehensive discussion of the functions of S1P and S1PRs in macrophage activation, the reader is referred to Weigert et al. [154].

*Mast Cells:* Mast cells participate in innate immunity and are generally found in epithelial and mucosal tissues [155]. Mast cells uniquely reach their mature differentiated state in the tissue in which they will reside, rather than in the BM from which the progenitors originate. They normally are not found in circulation. The destination tissue of mast cells is determined by specific cytokines, chemokines, integrins, and other growth factors [156]. For example, VCAM-1 directs mast cell progenitors out into the circulation [157], and CXCR2 helps direct them to the small intestine [158]. Two types of mast cells, mucosal or connective tissue cells, are found in epithelial and mucosal tissues.

The role of sphingolipids in mast cell activation is well established. S1P is known to be secreted by mast cells once they are activated and activation is inhibited in the presence of sphingosine and ceramide [159]. However, only a few studies have addressed the role of sphingolipids in mast cell differentiation. Mucosal mast cells produce tryptase, while connective tissue mast cells produce tryptase, as well as chymase and carboxypeptidase [160]. Interestingly, Price et al. reported that when cord blood-derived progenitor cells were cultured with stem cell factor alone, they differentiated into tryptase-only producing mast cells, indicating a mucosal phenotype. However, exposure of cord blood progenitor cells to S1P (which physiologically is encountered in circulation en route to the destination tissue) augmented mast cell differentiation and also influenced them to produce chymase in addition to tryptase, phenocopying the connective tissue/epithelial phenotype [161]. These observations suggest a role for S1P in the differentiation of mast cells and in the development of their epithelial phenotype. The same study also revealed that the stimulation of macrophages with S1P caused the release of IL-6; as IL-6 is known to promote the differentiation of mast cells [161], it is possible that a macrophage-mediated mechanism can also participate in the pro-differentiation function of S1P.

### 2.5. Lymphocytes: T-Cells, B-Cells, Natural Killer Cells

Lymphoid hematopoiesis diverges from other blood cell lineages at the progenitor level, with the traditional view being that those common lymphoid progenitors (CLPs) give rise to T-cells, B-cells, and natural killer cells [162,163,164,165]. CLPs originate in the BM and then proceed to initiate lymphoid cell development in the thymus. The thymus itself possesses no self-renewal capacity and thus relies on CLPs being seeded within it to give rise to the various lymphoid cells [166]. However, recruitment of CLPs to the thymus is not a continuous process and often depends on thymic demand [167,168]. It is unclear whether T-cell lineage commitment occurs before the CLPs reach the thymus, or upon entrance, and there is also evidence for extra-thymically originating T-cells [169,170]. Early lymphoid differentiation is still not fully understood, with contradicting evidence existing on the role of CLPs and their exclusive commitment to cells of the lymphoid lineage [171].

The role of sphingolipids in lymphoid differentiation is being actively examined. Sphingolipids’ role in lymphocyte differentiation was suggested after modulation of the S1P lyase, the activity of which controls the only known way to exit the sphingolipid pathway (Figure 2). Vogel et al. showed that systemic loss of S1P lyase leads to an expected increase of S1P in serum [172]. The increase of serum S1P led to depletion of T cells from the peripheral blood and to sequestration of mature T cells in the thymus and lymph nodes [172], corroborating earlier findings that lymphocytic departure from the thymus is dependent on S1P gradients and directly impacted by S1P lyase activity [83]. Additionally, complete S1P lyase knock out also caused hypocellularity of the thymus which was linked to increased apoptosis and impaired maturation at the intermediary steps of T-cell development [83].

These results were supported and expanded by another study by Weber et al. who reported on the role of S1P lyase in postnatal thymocyte development [173]. The study found that S1P lyase deficient mice showed discontinuation of lymphopoiesis beginning at two weeks after birth, specifically as a result of the deficiency in T-cell progenitors [173]. Additionally, increased ceramide levels were detected in the thymus of S1P lyase knock out mice (*SGPL1^−/−^*) which led to the increased apoptosis of thymocytes causing further depletion of T-cell progenitors [173].

As mentioned earlier, plasma S1P is essential for lymphocytic egress from lymphoid organs, via S1PR_1_ located on the surface of T-cells [85,174,175]. Importantly though, this process is not mediated by the pool of S1P complexed with apolipoprotein M (S1P-ApoM) [176,177]. On the other hand, S1P-ApoM (and not S1P complexed to albumin) was shown to curb lymphopoiesis as mice lacking ApoM presented with expanded proliferation of Lin^−^ Sca1^+^cKit^+^ hematopoietic progenitor cells and of CLPs [177]. Furthermore, the activation or overexpression of S1PR_1_ restored lymphopoiesis in the BM, suggesting that these changes were transduced via S1PR_1_-mediated signaling.

Additionally, changes in acid sphingomyelinase levels have been reported to alter the number of regulatory T cells in vitro and in vivo [178,179,180]. Specifically, the pharmacological and genetic inhibition of acid sphingomyelinase correlated with an increase in Foxp3^+^ regulatory T cells, possibly involving CD28 co-stimulation. These observations point to a negative role of acid sphingomyelinase on Foxp3^+^ regulatory T cells [178,179,180].

Limited research has addressed the roles of sphingolipids in the differentiation of B cells. One of the few studies implicates de novo ceramide synthesis in the differentiation of plasma cells [181]. The differentiation of plasma cells requires expansion of the endoplasmic reticulum (ER) in addition to that of secretory functions, and it is supported by the scaling up of both lipid and protein synthesis. Importantly, the expansion of the ER was largely reduced when de novo ceramide synthesis was blocked with fumonisin B1 [181]. Additionally, ceramide synthesis was stimulated by XBP-1 in its spliced form, a critical transcription factor regulating ER biogenesis in the context of ER stress. Furthermore, in line with a relevant function in quality control mechanisms for protein folding, the inhibition of ceramide synthesis prevented n-linked glycosylation; conversely, when an increase in de novo ceramide synthesis is observed, so is N-linked glycosylation. This relationship is not observed in resting B cells, rather only in those that are activated and differentiated into plasma cells, further supporting a role for ceramide synthesis in the process of plasma cell differentiation [181]. Of note, it has also been reported that B cells have a significantly lower ratio of dhCer/Cer in comparison to HSCs, however, the biological relevance of these findings is still unknown [55]. Interestingly, retinoic acid, which is known to modulate sphingolipid metabolism, regulates B cell differentiation, and retinoic acid together with α-galactosylceramide complementarily regulated B cell differentiation [182]. In addition to differentiation, there have been findings implicating sphingolipid-mediated signaling in B cell functions, such as S1P_4_ regulation of B cell migration [183].

A specific role for sphingolipids in the development of invariant natural killer T cells (*i*NKT) was also proposed [184]. Natural killer cells are a subset of T-cells that function as effectors of innate immunity as well as regulators of the immune response [185]. *i*NKT, in turn, are a subset of natural killer cells that mature in the thymus and mostly reside in the liver, where they comprise about 30% of the hematopoietic cells. Using CerS2 null mice, it was shown that very long chain sphingolipids in hematopoietic cells contribute to the maturation of *i*NKT in the thymus [184]. CerS2 is the enzyme that synthesizes dihydroceramide precursors utilized for the formation of sphingolipids with acyl chains made of 22 to 24 carbons (Figure 2), and CerS2 null mice are devoid of such sphingolipids. These mice also presented a significant decrease in *i*NKT cells in the thymus and the liver. Chimera BM transplantation experiments between CerS2 knock out and WT mice demonstrated the requirement of these sphingolipids for the generation of *i*NKT in the thymus, however, the molecular mechanism responsible for such modulation remains unknown.

## 3. Concluding Remarks

Hematopoietic stem cells are one of the major remedial components in BM transplantation and subsequently stem cell therapy. Dysregulation of the processes that govern HSC self-renewal and differentiation also leads to hematological diseases.

**Figure 5:**
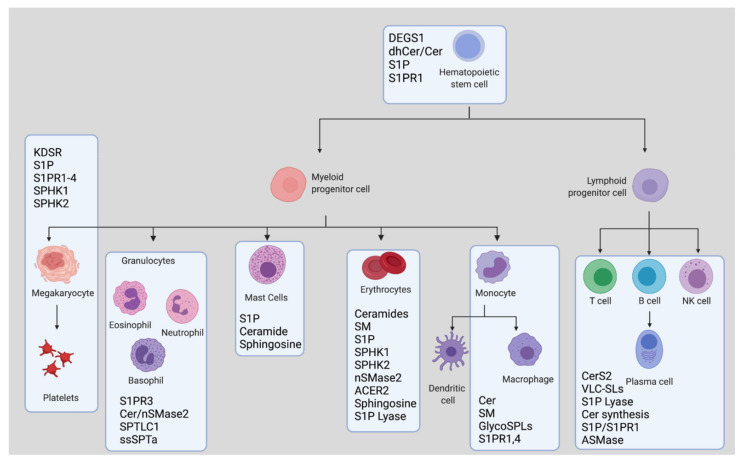
An Overview of Sphingolipid Involvement in Hematopoiesis and Lineage Commitment. Created with BioRender.com (accessed on 29 August 2021). S1P: sphingosine-1-phosphate, dhCer: dihydroceramide, Cer: ceramide, SM: sphingomyelin, VLC: very long chain, SL: sphingolipid, KDSR: 3 Ketodihydrosphingosine reductase, ACER: alkaline ceramidase, CerS: ceramide synthase, DEGS: delta 4-desaturase, SPHK: sphingosine kinase, S1PR: sphingosine-1-phosphate receptor, nSMase: neutral sphingomyelinase, ASMase: acid sphingomyelinase, SPTLC: serine palmitoyltransferase long base chain subunit, ssSPTa: small subunit of SPT.

In this review of the literature, we have discussed several functions for different sphingolipids and sphingolipid-metabolizing enzymes in the regulation of the distinct hematological lineage compartments. The involvement of specific components of the sphingolipid pathway in the different arms of lineage commitment discussed throughout the manuscript is summarized in the schematic (Figure 5).

The literature discussed herein not only highlights the emerging understanding of the roles and functions of sphingolipids in hematopoiesis but may provide a connection between altered sphingolipid metabolism and pathological conditions of the hematopoietic system, including hematological malignancies. Therefore, further understanding of the process by which stem/progenitor cells commit to the different lineages and further sphingolipid research in (patho)physiological conditions is vital to fully utilize and optimize metabolic tools to understand the mechanism of hematopoietic dysfunction and advance therapies.

## References

[1] Chao M.P., Seita J., Weissman I.L. (2008). Establishment of a normal hematopoietic and leukemia stem cell hierarchy. Cold Spring Harb. Symp. Quant. Biol..

[2] Metcalf D. (1984). The Hemopoietic Colony Stimulating Factors.

[3] Loughran S.J., Haas S., Wilkinson A.C., Klein A.M., Brand M. (2020). Lineage commitment of hematopoietic stem cells and progenitors: Insights from recent single cell and lineage tracing technologies. Exp. Hematol..

[4] Laurenti E., Gottgens B. (2018). From haematopoietic stem cells to complex differentiation landscapes. Nature.

[5] Takayama N., Murison A., Takayanagi S.I., Arlidge C., Zhou S., Garcia-Prat L., Chan-Seng-Yue M., Zandi S., Gan O.I., Boutzen H. (2021). The Transition from Quiescent to Activated States in Human Hematopoietic Stem Cells Is Governed by Dynamic 3D Genome Reorganization. Cell Stem Cell.

[6] Woolthuis C.M., Park C.Y. (2016). Hematopoietic stem/progenitor cell commitment to the megakaryocyte lineage. Blood.

[7] Carrelha J., Meng Y., Kettyle L.M., Luis T.C., Norfo R., Alcolea V., Boukarabila H., Grasso F., Gambardella A., Grover A. (2018). Hierarchically related lineage-restricted fates of multipotent haematopoietic stem cells. Nature.

[8] Notta F., Zandi S., Takayama N., Dobson S., Gan O.I., Wilson G., Kaufmann K.B., McLeod J., Laurenti E., Dunant C.F. (2016). Distinct routes of lineage development reshape the human blood hierarchy across ontogeny. Science.

[9] Adolfsson J., Borge O.J., Bryder D., Theilgaard-Monch K., Astrand-Grundstrom I., Sitnicka E., Sasaki Y., Jacobsen S.E. (2001). Upregulation of Flt3 expression within the bone marrow Lin(-)Sca1(+)c-kit(+) stem cell compartment is accompanied by loss of self-renewal capacity. Immunity.

[10] Pernes G., Flynn M.C., Lancaster G.I., Murphy A.J. (2019). Fat for fuel: Lipid metabolism in haematopoiesis. Clin. Transl. Immunol..

[11] Zhang Y., Gao S., Xia J., Liu F. (2018). Hematopoietic Hierarchy—An Updated Roadmap. Trends Cell Biol..

[12] Robb L. (2007). Cytokine receptors and hematopoietic differentiation. Oncogene.

[13] Rieger M.A., Schroeder T. (2012). Hematopoiesis. Cold Spring Harb. Perspect. Biol..

[14] Doulatov S., Notta F., Laurenti E., Dick J.E. (2012). Hematopoiesis: A human perspective. Cell Stem Cell.

[15] Dharampuriya P.R., Scapin G., Wong C., John Wagner K., Cillis J.L., Shah D.I. (2017). Tracking the origin, development, and differentiation of hematopoietic stem cells. Curr. Opin. Cell Biol..

[16] Hofer T., Rodewald H.R. (2018). Differentiation-based model of hematopoietic stem cell functions and lineage pathways. Blood.

[17] Ohta H., Sweeney E.A., Masamune A., Yatomi Y., Hakomori S., Igarashi Y. (1995). Induction of apoptosis by sphingosine in human leukemic HL-60 cells: A possible endogenous modulator of apoptotic DNA fragmentation occurring during phorbol ester-induced differentiation. Cancer Res..

[18] Hannun Y.A., Obeid L.M. (2018). Sphingolipids and their metabolism in physiology and disease. Nat. Rev. Mol. Cell Biol..

[19] Hannun Y.A., Obeid L.M. (2008). Principles of bioactive lipid signalling: Lessons from sphingolipids. Nat. Rev. Mol. Cell Biol..

[20] Ogretmen B. (2018). Sphingolipid metabolism in cancer signalling and therapy. Nat. Rev. Cancer.

[21] Futerman A.H., Riezman H. (2005). The ins and outs of sphingolipid synthesis. Trends Cell Biol..

[22] Spiegel S., Merrill A.H. (1996). Sphingolipid metabolism and cell growth regulation. FASEB J..

[23] Wang Y., Niu Y., Zhang Z., Gable K., Gupta S.D., Somashekarappa N., Han G., Zhao H., Myasnikov A.G., Kalathur R.C. (2021). Structural insights into the regulation of human serine palmitoyltransferase complexes. Nat. Struct. Mol. Biol..

[24] Li S., Xie T., Liu P., Wang L., Gong X. (2021). Structural insights into the assembly and substrate selectivity of human SPT-ORMDL3 complex. Nat. Struct. Mol. Biol..

[25] Hanada K. (2003). Serine palmitoyltransferase, a key enzyme of sphingolipid metabolism. Biochim. Biophys. Acta.

[26] Han G., Gupta S.D., Gable K., Niranjanakumari S., Moitra P., Eichler F., Brown R.H., Harmon J.M., Dunn T.M. (2009). Identification of small subunits of mammalian serine palmitoyltransferase that confer distinct acyl-CoA substrate specificities. Proc. Natl. Acad. Sci. USA.

[27] Hornemann T., Wei Y., von Eckardstein A. (2007). Is the mammalian serine palmitoyltransferase a high-molecular-mass complex?. Biochem. J..

[28] Zhao L., Spassieva S., Gable K., Gupta S.D., Shi L.Y., Wang J., Bielawski J., Hicks W.L., Krebs M.P., Naggert J. (2015). Elevation of 20-carbon long chain bases due to a mutation in serine palmitoyltransferase small subunit b results in neurodegeneration. Proc. Natl. Acad. Sci. USA.

[29] Harmon J.M., Bacikova D., Gable K., Gupta S.D., Han G., Sengupta N., Somashekarappa N., Dunn T.M. (2013). Topological and functional characterization of the ssSPTs, small activating subunits of serine palmitoyltransferase. J. Biol. Chem..

[30] Levy M., Futerman A.H. (2010). Mammalian ceramide synthases. IUBMB Life.

[31] Kihara A., Igarashi Y. (2004). FVT-1 is a mammalian 3-ketodihydrosphingosine reductase with an active site that faces the cytosolic side of the endoplasmic reticulum membrane. J. Biol. Chem..

[32] Michel C., van Echten-Deckert G., Rother J., Sandhoff K., Wang E., Merrill A.H. (1997). Characterization of ceramide synthesis. A dihydroceramide desaturase introduces the 4,5-trans-double bond of sphingosine at the level of dihydroceramide. J. Biol. Chem..

[33] Marggraf W.D., Kanfer J.N. (1984). The phosphorylcholine acceptor in the phosphatidylcholine:ceramide cholinephosphotransferase reaction. Is the enzyme a transferase or a hydrolase?. Biochim. Biophys. Acta.

[34] Vacaru A.M., van den Dikkenberg J., Ternes P., Holthuis J.C. (2013). Ceramide phosphoethanolamine biosynthesis in Drosophila is mediated by a unique ethanolamine phosphotransferase in the Golgi lumen. J. Biol. Chem..

[35] Malgat M., Maurice A., Baraud J. (1986). Sphingomyelin and ceramide-phosphoethanolamine synthesis by microsomes and plasma membranes from rat liver and brain. J. Lipid Res..

[36] Liu Y.Y., Hill R.A., Li Y.T. (2013). Ceramide glycosylation catalyzed by glucosylceramide synthase and cancer drug resistance. Adv. Cancer Res..

[37] Stoffel W., Taniguchi N., Honke K., Fukuda M., Clausen H., Furukawa K., Hart G.W., Kannagi R., Kawasaki T., Kinoshita T., Muramatsu T. (2002). GalCer Synthase (Ceramide Galactosyltransferase, CGT). Handbook of Glycosyltransferases and Related Genes.

[38] Sugiura M., Kono K., Liu H., Shimizugawa T., Minekura H., Spiegel S., Kohama T. (2002). Ceramide kinase, a novel lipid kinase. Molecular cloning and functional characterization. J. Biol. Chem..

[39] Senkal C.E., Salama M.F., Snider A.J., Allopenna J.J., Rana N.A., Koller A., Hannun Y.A., Obeid L.M. (2017). Ceramide Is Metabolized to Acylceramide and Stored in Lipid Droplets. Cell Metab..

[40] Ohno Y., Kamiyama N., Nakamichi S., Kihara A. (2017). PNPLA1 is a transacylase essential for the generation of the skin barrier lipid omega-O-acylceramide. Nat. Commun..

[41] El Bawab S., Mao C., Obeid L.M., Hannun Y.A. (2002). Ceramidases in the regulation of ceramide levels and function. Subcell Biochem..

[42] Maceyka M., Milstien S., Spiegel S. (2005). Sphingosine kinases, sphingosine-1-phosphate and sphingolipidomics. Prostaglandins Lipid Mediat..

[43] Serra M., Saba J.D. (2010). Sphingosine 1-phosphate lyase, a key regulator of sphingosine 1-phosphate signaling and function. Adv. Enzyme Regul..

[44] Rosen H., Stevens R.C., Hanson M., Roberts E., Oldstone M.B. (2013). Sphingosine-1-phosphate and its receptors: Structure, signaling, and influence. Annu. Rev. Biochem.

[45] Sanchez T., Hla T. (2004). Structural and functional characteristics of S1P receptors. J. Cell Biochem..

[46] Tsai H.C., Han M.H. (2016). Sphingosine-1-Phosphate (S1P) and S1P Signaling Pathway: Therapeutic Targets in Autoimmunity and Inflammation. Drugs.

[47] Olesch C., Ringel C., Brune B., Weigert A. (2017). Beyond Immune Cell Migration: The Emerging Role of the Sphingosine-1-phosphate Receptor S1PR4 as a Modulator of Innate Immune Cell Activation. Mediat. Inflamm..

[48] Graler M.H., Bernhardt G., Lipp M. (1999). A lymphoid tissue-specific receptor, EDG6, with potential immune modulatory functions mediated by extracellular lysophospholipids. Curr. Top. Microbiol. Immunol..

[49] Graler M.H., Bernhardt G., Lipp M. (1998). EDG6, a novel G-protein-coupled receptor related to receptors for bioactive lysophospholipids, is specifically expressed in lymphoid tissue. Genomics.

[50] Evangelisti C., Evangelisti C., Buontempo F., Lonetti A., Orsini E., Chiarini F., Barata J.T., Pyne S., Pyne N.J., Martelli A.M. (2016). Therapeutic potential of targeting sphingosine kinases and sphingosine 1-phosphate in hematological malignancies. Leukemia.

[51] Kitatani K., Taniguchi M., Okazaki T. (2015). Role of Sphingolipids and Metabolizing Enzymes in Hematological Malignancies. Mol. Cells.

[52] Moorthi S., Luberto C., Hannun Y.A., Luberto C., Mao C., Obeid L.M. (2015). Role of Sphingolipids in Hematological Malignancies: Myeloproliferative Disorders. Bioactive Sphingolipids in Cancer Biology and Therapy.

[53] Sawai H., Taniguchi M., Okazaki T., Hannun Y.A., Luberto C., Mao C., Obeid L.M. (2015). Role of Sphingolipids in Hematological Malignancies: Lymphoproliferative Disorders. Bioactive Sphingolipids in Cancer Biology and Therapy.

[54] Maguer-Satta V., Oostendorp R., Reid D., Eaves C.J. (2000). Evidence that ceramide mediates the ability of tumor necrosis factor to modulate primitive human hematopoietic cell fates. Blood.

[55] Xie S.Z., Garcia-Prat L., Voisin V., Ferrari R., Gan O.I., Wagenblast E., Kaufmann K.B., Zeng A.G.X., Takayanagi S.I., Patel I. (2019). Sphingolipid Modulation Activates Proteostasis Programs to Govern Human Hematopoietic Stem Cell Self-Renewal. Cell Stem Cell.

[56] Golan K., Vagima Y., Ludin A., Itkin T., Cohen-Gur S., Kalinkovich A., Kollet O., Kim C., Schajnovitz A., Ovadya Y. (2012). S1P promotes murine progenitor cell egress and mobilization via S1P1-mediated ROS signaling and SDF-1 release. Blood.

[57] Ratajczak M.Z., Lee H., Wysoczynski M., Wan W., Marlicz W., Laughlin M.J., Kucia M., Janowska-Wieczorek A., Ratajczak J. (2010). Novel insight into stem cell mobilization-plasma sphingosine-1-phosphate is a major chemoattractant that directs the egress of hematopoietic stem progenitor cells from the bone marrow and its level in peripheral blood increases during mobilization due to activation of complement cascade/membrane attack complex. Leukemia.

[58] Juarez J.G., Harun N., Thien M., Welschinger R., Baraz R., Pena A.D., Pitson S.M., Rettig M., DiPersio J.F., Bradstock K.F. (2012). Sphingosine-1-phosphate facilitates trafficking of hematopoietic stem cells and their mobilization by CXCR4 antagonists in mice. Blood.

[59] Brinkmann V., Davis M.D., Heise C.E., Albert R., Cottens S., Hof R., Bruns C., Prieschl E., Baumruker T., Hiestand P. (2002). The immune modulator FTY720 targets sphingosine 1-phosphate receptors. J. Biol. Chem..

[60] Graler M.H., Goetzl E.J. (2004). The immunosuppressant FTY720 down-regulates sphingosine 1-phosphate G-protein-coupled receptors. FASEB J..

[61] Sugiyama T., Kohara H., Noda M., Nagasawa T. (2006). Maintenance of the hematopoietic stem cell pool by CXCL12-CXCR4 chemokine signaling in bone marrow stromal cell niches. Immunity.

[62] Nie Y., Han Y.C., Zou Y.R. (2008). CXCR4 is required for the quiescence of primitive hematopoietic cells. J. Exp. Med..

[63] Rosu-Myles M., Gallacher L., Murdoch B., Hess D.A., Keeney M., Kelvin D., Dale L., Ferguson S.S., Wu D., Fellows F. (2000). The human hematopoietic stem cell compartment is heterogeneous for CXCR4 expression. Proc. Natl. Acad. Sci. USA.

[64] Massberg S., Schaerli P., Knezevic-Maramica I., Kollnberger M., Tubo N., Moseman E.A., Huff I.V., Junt T., Wagers A.J., Mazo I.B. (2007). Immunosurveillance by hematopoietic progenitor cells trafficking through blood, lymph, and peripheral tissues. Cell.

[65] Liu J., Zhao J., Lee J.F., Gartung A., Jawadi H., Zhang W., Lominadze D., Lee M.J. (2013). 3-amino-4-(3-hexylphenylamino)-4-oxobutyl phosphonic acid (W146), a Selective Antagonist of Sphingosine-1-phospahte Receptor Subtype 1, Enhances AMD3100-stimulated Mobilization of Hematopoietic Stem Progenitor Cells in Animals. J. Biochem. Pharmacol. Res..

[66] Gregory C.J., Eaves A.C. (1977). Human marrow cells capable of erythropoietic differentiation in vitro: Definition of three erythroid colony responses. Blood.

[67] Koury M.J., Bondurant M.C. (1990). Control of red cell production: The roles of programmed cell death (apoptosis) and erythropoietin. Transfusion.

[68] Koury M.J., Bondurant M.C. (1992). The molecular mechanism of erythropoietin action. Eur. J. Biochem..

[69] Palis J. (2014). Primitive and definitive erythropoiesis in mammals. Front. Physiol..

[70] Moras M., Lefevre S.D., Ostuni M.A. (2017). From Erythroblasts to Mature Red Blood Cells: Organelle Clearance in Mammals. Front. Physiol..

[71] Clayton R.B., Cooper J.M., Curstedt T., Sjovall J., Borsook H., Chin J., Schwarz A. (1974). Stimulation of erythroblast maturation in vitro by sphingolipids. J. Lipid Res..

[72] Scaro J.L., Miranda C., Martin B.M., Carrera M. (1982). Effects of sphingolipids on erythroblastic maturation in the mouse. Experientia.

[73] Dallalio G., North M., Worden B.D., Means R.T. (1999). Inhibition of human erythroid colony formation by ceramide. Exp. Hematol..

[74] Orsini M., Chateauvieux S., Rhim J., Gaigneaux A., Cheillan D., Christov C., Dicato M., Morceau F., Diederich M. (2019). Sphingolipid-mediated inflammatory signaling leading to autophagy inhibition converts erythropoiesis to myelopoiesis in human hematopoietic stem/progenitor cells. Cell Death Differ..

[75] Yang C., Hashimoto M., Lin Q.X.X., Tan D.Q., Suda T. (2019). Sphingosine-1-phosphate signaling modulates terminal erythroid differentiation through the regulation of mitophagy. Exp. Hematol..

[76] Sandoval H., Thiagarajan P., Dasgupta S.K., Schumacher A., Prchal J.T., Chen M., Wang J. (2008). Essential role for Nix in autophagic maturation of erythroid cells. Nature.

[77] Wu L., Xu W., Xu L., Kong Q., Fang J. (2017). Mitophagy is increased during erythroid differentiation in beta-thalassemia. Int. J. Hematol..

[78] Xiong Y., Yang P., Proia R.L., Hla T. (2014). Erythrocyte-derived sphingosine 1-phosphate is essential for vascular development. J. Clin. Investig..

[79] Rusten L.S., Jacobsen S.E. (1995). Tumor necrosis factor (TNF)-alpha directly inhibits human erythropoiesis in vitro: Role of p55 and p75 TNF receptors. Blood.

[80] Jacobs-Helber S.M., Roh K.H., Bailey D., Dessypris E.N., Ryan J.J., Chen J., Wickrema A., Barber D.L., Dent P., Sawyer S.T. (2003). Tumor necrosis factor-alpha expressed constitutively in erythroid cells or induced by erythropoietin has negative and stimulatory roles in normal erythropoiesis and erythroleukemia. Blood.

[81] Zhang J., Wu K., Xiao X., Liao J., Hu Q., Chen H., Liu J., An X. (2015). Autophagy as a regulatory component of erythropoiesis. Int. J. Mol. Sci..

[82] Pappu R., Schwab S.R., Cornelissen I., Pereira J.P., Regard J.B., Xu Y., Camerer E., Zheng Y.W., Huang Y., Cyster J.G. (2007). Promotion of lymphocyte egress into blood and lymph by distinct sources of sphingosine-1-phosphate. Science.

[83] Schwab S.R., Pereira J.P., Matloubian M., Xu Y., Huang Y., Cyster J.G. (2005). Lymphocyte sequestration through S1P lyase inhibition and disruption of S1P gradients. Science.

[84] Obinata H., Hla T. (2019). Sphingosine 1-phosphate and inflammation. Int. Immunol..

[85] Matloubian M., Lo C.G., Cinamon G., Lesneski M.J., Xu Y., Brinkmann V., Allende M.L., Proia R.L., Cyster J.G. (2004). Lymphocyte egress from thymus and peripheral lymphoid organs is dependent on S1P receptor 1. Nature.

[86] Ito K., Anada Y., Tani M., Ikeda M., Sano T., Kihara A., Igarashi Y. (2007). Lack of sphingosine 1-phosphate-degrading enzymes in erythrocytes. Biochem. Biophys. Res. Commun..

[87] Nguyen T.Q., Vu T.M., Tukijan F., Muralidharan S., Foo J.C., Chin J.F.L., Hasan Z., Torta F., Nguyen L.N. (2020). Erythrocytes efficiently utilize exogenous sphingosines for S1P synthesis and export via Mfsd2b. J. Biol. Chem..

[88] Li F., Xu R., Low B.E., Lin C.L., Garcia-Barros M., Schrandt J., Mileva I., Snider A., Luo C.K., Jiang X.C. (2018). Alkaline ceramidase 2 is essential for the homeostasis of plasma sphingoid bases and their phosphates. FASEB J..

[89] Vu T.M., Ishizu A.N., Foo J.C., Toh X.R., Zhang F., Whee D.M., Torta F., Cazenave-Gassiot A., Matsumura T., Kim S. (2017). Mfsd2b is essential for the sphingosine-1-phosphate export in erythrocytes and platelets. Nature.

[90] Kobayashi N., Kawasaki-Nishi S., Otsuka M., Hisano Y., Yamaguchi A., Nishi T. (2018). MFSD2B is a sphingosine 1-phosphate transporter in erythroid cells. Sci. Rep..

[91] Kurano M., Nishikawa M., Kuma H., Jona M., Yatomi Y. (2017). Involvement of Band3 in the efflux of sphingosine 1-phosphate from erythrocytes. PLoS ONE.

[92] Hanel P., Andreani P., Graler M.H. (2007). Erythrocytes store and release sphingosine 1-phosphate in blood. FASEB J..

[93] Bode C., Sensken S.C., Peest U., Beutel G., Thol F., Levkau B., Li Z., Bittman R., Huang T., Tolle M. (2010). Erythrocytes serve as a reservoir for cellular and extracellular sphingosine 1-phosphate. J. Cell Biochem..

[94] Sutter I., Park R., Othman A., Rohrer L., Hornemann T., Stoffel M., Devuyst O., von Eckardstein A. (2014). Apolipoprotein M modulates erythrocyte efflux and tubular reabsorption of sphingosine-1-phosphate. J. Lipid Res..

[95] Sun K., Zhang Y., D’Alessandro A., Nemkov T., Song A., Wu H., Liu H., Adebiyi M., Huang A., Wen Y.E. (2016). Sphingosine-1-phosphate promotes erythrocyte glycolysis and oxygen release for adaptation to high-altitude hypoxia. Nat. Commun..

[96] Xie T., Chen C., Peng Z., Brown B.C., Reisz J.A., Xu P., Zhou Z., Song A., Zhang Y., Bogdanov M.V. (2020). Erythrocyte Metabolic Reprogramming by Sphingosine 1-Phosphate in Chronic Kidney Disease and Therapies. Circ. Res..

[97] Akunov A., Sydykov A., Toktash T., Doolotova A., Sarybaev A. (2018). Hemoglobin Changes After Long-Term Intermittent Work at High Altitude. Front. Physiol..

[98] Huang N.J., Lin Y.C., Lin C.Y., Pishesha N., Lewis C.A., Freinkman E., Farquharson C., Millan J.L., Lodish H. (2018). Enhanced phosphocholine metabolism is essential for terminal erythropoiesis. Blood.

[99] Leonard C., Conrard L., Guthmann M., Pollet H., Carquin M., Vermylen C., Gailly P., Van Der Smissen P., Mingeot-Leclercq M.P., Tyteca D. (2017). Contribution of plasma membrane lipid domains to red blood cell (re)shaping. Sci. Rep..

[100] Conrard L., Stommen A., Cloos A.S., Steinkuhler J., Dimova R., Pollet H., Tyteca D. (2018). Spatial Relationship and Functional Relevance of Three Lipid Domain Populations at the Erythrocyte Surface. Cell Physiol. Biochem..

[101] Patel S.R., Hartwig J.H., Italiano J.E. (2005). The biogenesis of platelets from megakaryocyte proplatelets. J. Clin. Investig..

[102] Machlus K.R., Italiano J.E. (2013). The incredible journey: From megakaryocyte development to platelet formation. J. Cell Biol..

[103] Zhang L., Orban M., Lorenz M., Barocke V., Braun D., Urtz N., Schulz C., von Bruhl M.L., Tirniceriu A., Gaertner F. (2012). A novel role of sphingosine 1-phosphate receptor S1pr1 in mouse thrombopoiesis. J. Exp. Med..

[104] Bunting S., Widmer R., Lipari T., Rangell L., Steinmetz H., Carver-Moore K., Moore M.W., Keller G.A., de Sauvage F.J. (1997). Normal platelets and megakaryocytes are produced in vivo in the absence of thrombopoietin. Blood.

[105] Hla T., Maciag T. (1990). An abundant transcript induced in differentiating human endothelial cells encodes a polypeptide with structural similarities to G-protein-coupled receptors. J. Biol. Chem..

[106] Okazaki H., Ishizaka N., Sakurai T., Kurokawa K., Goto K., Kumada M., Takuwa Y. (1993). Molecular cloning of a novel putative G protein-coupled receptor expressed in the cardiovascular system. Biochem. Biophys. Res. Commun..

[107] Lee M.J., Van Brocklyn J.R., Thangada S., Liu C.H., Hand A.R., Menzeleev R., Spiegel S., Hla T. (1998). Sphingosine-1-phosphate as a ligand for the G protein-coupled receptor EDG-1. Science.

[108] Yamaguchi F., Tokuda M., Hatase O., Brenner S. (1996). Molecular cloning of the novel human G protein-coupled receptor (GPCR) gene mapped on chromosome 9. Biochem. Biophys. Res. Commun..

[109] Im D.S., Heise C.E., Ancellin N., O’Dowd B.F., Shei G.J., Heavens R.P., Rigby M.R., Hla T., Mandala S., McAllister G. (2000). Characterization of a novel sphingosine 1-phosphate receptor, Edg-8. J. Biol. Chem..

[110] Spiegel S. (2000). Sphingosine 1-phosphate: A ligand for the EDG-1 family of G-protein-coupled receptors. Ann. N. Y. Acad. Sci..

[111] Golfier S., Kondo S., Schulze T., Takeuchi T., Vassileva G., Achtman A.H., Graler M.H., Abbondanzo S.J., Wiekowski M., Kremmer E. (2010). Shaping of terminal megakaryocyte differentiation and proplatelet development by sphingosine-1-phosphate receptor S1P4. FASEB J..

[112] Niazi H., Zoghdani N., Couty L., Leuci A., Nitzsche A., Allende M.L., Mariko B., Ishaq R., Aslan Y., Becker P.H. (2019). Murine platelet production is suppressed by S1P release in the hematopoietic niche, not facilitated by blood S1P sensing. Blood Adv..

[113] Zhang L., Urtz N., Gaertner F., Legate K.R., Petzold T., Lorenz M., Mazharian A., Watson S.P., Massberg S. (2013). Sphingosine kinase 2 (Sphk2) regulates platelet biogenesis by providing intracellular sphingosine 1-phosphate (S1P). Blood.

[114] Takeichi T., Torrelo A., Lee J.Y.W., Ohno Y., Lozano M.L., Kihara A., Liu L., Yasuda Y., Ishikawa J., Murase T. (2017). Biallelic Mutations in KDSR Disrupt Ceramide Synthesis and Result in a Spectrum of Keratinization Disorders Associated with Thrombocytopenia. J. Investig. Dermatol..

[115] Bariana T.K., Labarque V., Heremans J., Thys C., De Reys M., Greene D., Jenkins B., Grassi L., Seyres D., Burden F. (2019). Sphingolipid dysregulation due to lack of functional KDSR impairs proplatelet formation causing thrombocytopenia. Haematologica.

[116] Xie S.Z., Kaufmann K.B., Wang W., Chan-Seng-Yue M., Gan O.I., Laurenti E., Garcia-Prat L., Takayanagi S.I., Ng S.W.K., Xu C. (2021). Sphingosine-1-phosphate receptor 3 potentiates inflammatory programs in normal and leukemia stem cells to promote differentiation. Blood Cancer Discov..

[117] Yatomi Y., Ruan F., Hakomori S., Igarashi Y. (1995). Sphingosine-1-phosphate: A platelet-activating sphingolipid released from agonist-stimulated human platelets. Blood.

[118] Murate T., Banno Y., T-Koizumi K., Watanabe K., Mori N., Wada A., Igarashi Y., Takagi A., Kojima T., Asano H. (2001). Cell type-specific localization of sphingosine kinase 1a in human tissues. J. Histochem. Cytochem..

[119] Urtz N., Gaertner F., von Bruehl M.L., Chandraratne S., Rahimi F., Zhang L., Orban M., Barocke V., Beil J., Schubert I. (2015). Sphingosine 1-Phosphate Produced by Sphingosine Kinase 2 Intrinsically Controls Platelet Aggregation In Vitro and In Vivo. Circ. Res..

[120] Yatomi Y., Yamamura S., Hisano N., Nakahara K., Igarashi Y., Ozaki Y. (2004). Sphingosine 1-phosphate breakdown in platelets. J. Biochem..

[121] Tani M., Sano T., Ito M., Igarashi Y. (2005). Mechanisms of sphingosine and sphingosine 1-phosphate generation in human platelets. J. Lipid Res..

[122] Venkataraman K., Lee Y.M., Michaud J., Thangada S., Ai Y., Bonkovsky H.L., Parikh N.S., Habrukowich C., Hla T. (2008). Vascular endothelium as a contributor of plasma sphingosine 1-phosphate. Circ. Res..

[123] Gazit S.L., Mariko B., Therond P., Decouture B., Xiong Y., Couty L., Bonnin P., Baudrie V., Le Gall S.M., Dizier B. (2016). Platelet and Erythrocyte Sources of S1P Are Redundant for Vascular Development and Homeostasis, but Both Rendered Essential After Plasma S1P Depletion in Anaphylactic Shock. Circ. Res..

[124] Yatomi Y., Yamamura S., Ruan F., Igarashi Y. (1997). Sphingosine 1-phosphate induces platelet activation through an extracellular action and shares a platelet surface receptor with lysophosphatidic acid. J. Biol. Chem..

[125] Aoki S., Osada M., Kaneko M., Ozaki Y., Yatomi Y. (2007). Fluid shear stress enhances the sphingosine 1-phosphate responses in cell-cell interactions between platelets and endothelial cells. Biochem. Biophys. Res. Commun..

[126] Ono Y., Kurano M., Ohkawa R., Yokota H., Igarashi K., Aoki J., Tozuka M., Yatomi Y. (2013). Sphingosine 1-phosphate release from platelets during clot formation: Close correlation between platelet count and serum sphingosine 1-phosphate concentration. Lipids Health Dis..

[127] Vogt K., Mahajan-Thakur S., Wolf R., Broderdorf S., Vogel C., Bohm A., Ritter C.A., Graler M., Oswald S., Greinacher A. (2018). Release of Platelet-Derived Sphingosine-1-Phosphate Involves Multidrug Resistance Protein 4 (MRP4/ABCC4) and Is Inhibited by Statins. Thromb. Haemost..

[128] Decouture B., Becker P.H., Therond P., Gaussem P., Bachelot-Loza C. (2018). Evidence that MRP4 is Only Partly Involved in S1P Secretion during Platelet Activation. Thromb. Haemost..

[129] Akashi K., Traver D., Miyamoto T., Weissman I.L. (2000). A clonogenic common myeloid progenitor that gives rise to all myeloid lineages. Nature.

[130] Zon L.I., Yamaguchi Y., Yee K., Albee E.A., Kimura A., Bennett J.C., Orkin S.H., Ackerman S.J. (1993). Expression of mRNA for the GATA-binding proteins in human eosinophils and basophils: Potential role in gene transcription. Blood.

[131] Drissen R., Thongjuea S., Theilgaard-Monch K., Nerlov C. (2019). Identification of two distinct pathways of human myelopoiesis. Sci. Immunol..

[132] Parthibane V., Acharya D., Srideshikan S.M., Lin J., Myerscough D.G., Abimannan T., Vijaykrishna N., Blankenberg D., Bondada L., Klarmann K.D. (2019). Sptlc1 is essential for myeloid differentiation and hematopoietic homeostasis. Blood Adv..

[133] Parthibane V., Lin J., Acharya D., Abimannan T., Srideshikan S.M., Klarmann K., Yang A., Soheilian F., Nagashima K., Fox S.D. (2021). SSSPTA is essential for serine palmitoyltransferase function during development and hematopoiesis. J. Biol. Chem..

[134] Nojiri H., Takaku F., Tetsuka T., Motoyoshi K., Miura Y., Saito M. (1984). Characteristic expression of glycosphingolipid profiles in the bipotential cell differentiation of human promyelocytic leukemia cell line HL-60. Blood.

[135] Ryan J.L., Yohe H.C., Malech H.L. (1985). Changes in membrane gangliosides: Differentiation of human and murine monocytic cells. Yale J. Biol Med..

[136] Delannoy C.P., Rombouts Y., Groux-Degroote S., Holst S., Coddeville B., Harduin-Lepers A., Wuhrer M., Elass-Rochard E., Guerardel Y. (2017). Glycosylation Changes Triggered by the Differentiation of Monocytic THP-1 Cell Line into Macrophages. J. Proteome Res..

[137] Momoi T., Shinmoto M., Kasuya J., Senoo H., Suzuki Y. (1986). Activation of CMP-N-acetylneuraminic acid:lactosylceramide sialyltransferase during the differentiation of HL-60 cells induced by 12-O-tetradecanoylphorbol-13-acetate. J. Biol. Chem..

[138] Senoo H., Momoi T. (1985). The differentiation of HL-60 cells in the synthetic medium induced by GM3 ganglioside. Biosci. Rep..

[139] Nojiri H., Takaku F., Terui Y., Miura Y., Saito M. (1986). Ganglioside GM3: An acidic membrane component that increases during macrophage-like cell differentiation can induce monocytic differentiation of human myeloid and monocytoid leukemic cell lines HL-60 and U937. Proc. Natl. Acad. Sci. USA.

[140] Kiguchi K., Henning-Chubb C., Huberman E. (1993). Glycosphingolipid patterns in human promyelocytic HL-60 leukemia cells susceptible or resistant to differentiation induction by phorbol 12-myristate 13-acetate. Biochim. Biophys. Acta.

[141] Gracheva E.V., Samovilova N.N., Golovanova N.K., Kashirina S.V., Shevelev A., Rybalkin I., Gurskaya T., Vlasik T.N., Andreeva E.R., Prokazova N.V. (2009). Enhancing of GM3 synthase expression during differentiation of human blood monocytes into macrophages as in vitro model of GM3 accumulation in atherosclerotic lesion. Mol. Cell Biochem..

[142] Akagawa K.S., Momoi T., Nagai Y., Tokunaga T. (1981). Appearance of asialo GM1 glycosphingolipid on the cell surface during lymphokine-induced differentiation of M1 cells. FEBS Lett..

[143] Moreno-Altamirano M.M., Aguilar-Carmona I., Sanchez-Garcia F.J. (2007). Expression of GM1, a marker of lipid rafts, defines two subsets of human monocytes with differential endocytic capacity and lipopolysaccharide responsiveness. Immunology.

[144] Aida J., Higuchi S., Hasegawa Y., Nagano-Ito M., Hirabayashi Y., Banba A., Shimizu T., Kikuchi A., Saga M., Ichikawa S. (2011). Up-regulation of ceramide glucosyltransferase during the differentiation of U937 cells. J. Biochem..

[145] Kan C.C., Kolesnick R.N. (1992). A synthetic ceramide analog, D-threo-1-phenyl-2-decanoylamino-3-morpholino-1-propanol, selectively inhibits adherence during macrophage differentiation of human leukemia cells. J. Biol. Chem..

[146] Dressler K.A., Kan C.C., Kolesnick R.N. (1991). Sphingomyelin synthesis is involved in adherence during macrophage differentiation of HL-60 cells. J. Biol. Chem..

[147] Yamamoto H., Naito Y., Okano M., Kanazawa T., Takematsu H., Kozutsumi Y. (2011). Sphingosylphosphorylcholine and lysosulfatide have inverse regulatory functions in monocytic cell differentiation into macrophages. Arch. Biochem. Biophys..

[148] Rovina P., Graf C., Bornancin F. (2010). Modulation of ceramide metabolism in mouse primary macrophages. Biochem. Biophys. Res. Commun..

[149] Okazaki T., Bielawska A., Bell R.M., Hannun Y.A. (1990). Role of ceramide as a lipid mediator of 1 alpha,25-dihydroxyvitamin D3-induced HL-60 cell differentiation. J. Biol. Chem..

[150] Wallner S., Grandl M., Liebisch G., Peer M., Orso E., Sigruner A., Sobota A., Schmitz G. (2016). oxLDL and eLDL Induced Membrane Microdomains in Human Macrophages. PLoS ONE.

[151] Lee H., Liao J.J., Graeler M., Huang M.C., Goetzl E.J. (2002). Lysophospholipid regulation of mononuclear phagocytes. Biochim. Biophys. Acta.

[152] Hohenhaus D.M., Schaale K., Le Cao K.A., Seow V., Iyer A., Fairlie D.P., Sweet M.J. (2013). An mRNA atlas of G protein-coupled receptor expression during primary human monocyte/macrophage differentiation and lipopolysaccharide-mediated activation identifies targetable candidate regulators of inflammation. Immunobiology.

[153] Muller J., von Bernstorff W., Heidecke C.D., Schulze T. (2017). Differential S1P Receptor Profiles on M1- and M2-Polarized Macrophages Affect Macrophage Cytokine Production and Migration. Biomed. Res. Int..

[154] Weigert A., Olesch C., Brune B. (2019). Sphingosine-1-Phosphate and Macrophage Biology-How the Sphinx Tames the Big Eater. Front. Immunol..

[155] Krystel-Whittemore M., Dileepan K.N., Wood J.G. (2015). Mast Cell: A Multi-Functional Master Cell. Front. Immunol..

[156] Collington S.J., Williams T.J., Weller C.L. (2011). Mechanisms underlying the localisation of mast cells in tissues. Trends Immunol..

[157] Boyce J.A., Mellor E.A., Perkins B., Lim Y.C., Luscinskas F.W. (2002). Human mast cell progenitors use alpha4-integrin, VCAM-1, and PSGL-1 E-selectin for adhesive interactions with human vascular endothelium under flow conditions. Blood.

[158] Abonia J.P., Austen K.F., Rollins B.J., Joshi S.K., Flavell R.A., Kuziel W.A., Koni P.A., Gurish M.F. (2005). Constitutive homing of mast cell progenitors to the intestine depends on autologous expression of the chemokine receptor CXCR2. Blood.

[159] Olivera A., Rivera J. (2005). Sphingolipids and the balancing of immune cell function: Lessons from the mast cell. J. Immunol..

[160] Irani A.A., Schechter N.M., Craig S.S., DeBlois G., Schwartz L.B. (1986). Two types of human mast cells that have distinct neutral protease compositions. Proc. Natl. Acad. Sci. USA.

[161] Price M.M., Kapitonov D., Allegood J., Milstien S., Oskeritzian C.A., Spiegel S. (2009). Sphingosine-1-phosphate induces development of functionally mature chymase-expressing human mast cells from hematopoietic progenitors. FASEB J..

[162] Kondo M. (2010). Lymphoid and myeloid lineage commitment in multipotent hematopoietic progenitors. Immunol. Rev..

[163] Kondo M., Scherer D.C., Miyamoto T., King A.G., Akashi K., Sugamura K., Weissman I.L. (2000). Cell-fate conversion of lymphoid-committed progenitors by instructive actions of cytokines. Nature.

[164] Kondo M., Weissman I.L., Akashi K. (1997). Identification of clonogenic common lymphoid progenitors in mouse bone marrow. Cell.

[165] Igarashi H., Gregory S.C., Yokota T., Sakaguchi N., Kincade P.W. (2002). Transcription from the RAG1 locus marks the earliest lymphocyte progenitors in bone marrow. Immunity.

[166] Wallis V.J., Leuchars E., Chwalinski S., Davies A.J. (1975). On the sparse seeding of bone marrow and thymus in radiation chimaeras. Transplantation.

[167] Foss D.L., Donskoy E., Goldschneider I. (2001). The importation of hematogenous precursors by the thymus is a gated phenomenon in normal adult mice. J. Exp. Med..

[168] Donskoy E., Foss D., Goldschneider I. (2003). Gated importation of prothymocytes by adult mouse thymus is coordinated with their periodic mobilization from bone marrow. J. Immunol..

[169] Rocha B. (1990). Characterization of V beta-bearing cells in athymic (nu/nu) mice suggests an extrathymic pathway for T cell differentiation. Eur. J. Immunol..

[170] Rocha B. (2007). The extrathymic T-cell differentiation in the murine gut. Immunol. Rev..

[171] Masuda K., Itoi M., Amagai T., Minato N., Katsura Y., Kawamoto H. (2005). Thymic anlage is colonized by progenitors restricted to T, NK, and dendritic cell lineages. J. Immunol..

[172] Vogel P., Donoviel M.S., Read R., Hansen G.M., Hazlewood J., Anderson S.J., Sun W., Swaffield J., Oravecz T. (2009). Incomplete inhibition of sphingosine 1-phosphate lyase modulates immune system function yet prevents early lethality and non-lymphoid lesions. PLoS ONE.

[173] Weber C., Krueger A., Munk A., Bode C., Van Veldhoven P.P., Graler M.H. (2009). Discontinued postnatal thymocyte development in sphingosine 1-phosphate-lyase-deficient mice. J. Immunol..

[174] Thangada S., Khanna K.M., Blaho V.A., Oo M.L., Im D.S., Guo C., Lefrancois L., Hla T. (2010). Cell-surface residence of sphingosine 1-phosphate receptor 1 on lymphocytes determines lymphocyte egress kinetics. J. Exp. Med..

[175] Maeda Y., Seki N., Sato N., Sugahara K., Chiba K. (2010). Sphingosine 1-phosphate receptor type 1 regulates egress of mature T cells from mouse bone marrow. Int. Immunol..

[176] Pyne N.J., Pyne S. (2017). Sphingosine 1-Phosphate Receptor 1 Signaling in Mammalian Cells. Molecules.

[177] Blaho V.A., Galvani S., Engelbrecht E., Liu C., Swendeman S.L., Kono M., Proia R.L., Steinman L., Han M.H., Hla T. (2015). HDL-bound sphingosine-1-phosphate restrains lymphopoiesis and neuroinflammation. Nature.

[178] Wiese T., Dennstadt F., Hollmann C., Stonawski S., Wurst C., Fink J., Gorte E., Mandasari P., Domschke K., Hommers L. (2021). Inhibition of acid sphingomyelinase increases regulatory T cells in humans. Brain Commun..

[179] Zhou Y., Salker M.S., Walker B., Munzer P., Borst O., Gawaz M., Gulbins E., Singh Y., Lang F. (2016). Acid Sphingomyelinase (ASM) is a Negative Regulator of Regulatory T Cell (Treg) Development. Cell Physiol. Biochem..

[180] Hollmann C., Werner S., Avota E., Reuter D., Japtok L., Kleuser B., Gulbins E., Becker K.A., Schneider-Schaulies J., Beyersdorf N. (2016). Inhibition of Acid Sphingomyelinase Allows for Selective Targeting of CD4+ Conventional versus Foxp3+ Regulatory T Cells. J. Immunol..

[181] Goldfinger M., Laviad E.L., Hadar R., Shmuel M., Dagan A., Park H., Merrill A.H., Ringel I., Futerman A.H., Tirosh B. (2009). De novo ceramide synthesis is required for N-linked glycosylation in plasma cells. J. Immunol..

[182] Chen Q., Mosovsky K.L., Ross A.C. (2013). Retinoic acid and alpha-galactosylceramide regulate the expression of costimulatory receptors and transcription factors responsible for B cell activation and differentiation. Immunobiology.

[183] Kleinwort A., Luhrs F., Heidecke C.D., Lipp M., Schulze T. (2018). S1P Signalling Differentially Affects Migration of Peritoneal B Cell Populations In Vitro and Influences the Production of Intestinal IgA In Vivo. Int. J. Mol. Sci..

[184] Saroha A., Pewzner-Jung Y., Ferreira N.S., Sharma P., Jouan Y., Kelly S.L., Feldmesser E., Merrill A.H., Trottein F., Paget C. (2017). Critical Role for Very-Long Chain Sphingolipids in Invariant Natural Killer T Cell Development and Homeostasis. Front. Immunol..

[185] Vivier E., Tomasello E., Baratin M., Walzer T., Ugolini S. (2008). Functions of natural killer cells. Nat. Immunol..

